# A direct multiplex isothermal amplification-reverse dot blot hybridization system for β-thalassemia diagnosis

**DOI:** 10.1007/s00277-025-06711-5

**Published:** 2025-11-18

**Authors:** Chao Ye, Xiaoxing Zhou, Yan Wei, Yilian Zhao, Mengru Xie, Xinchu Liu, Jinghui Ma, Jilin Qing, Zhizhong Chen

**Affiliations:** 1 Joint Inspection Center of Precision Medicine, The People’s Hospital of Guangxi Zhuang Autonomous Region and Guangxi Academy of Medical Sciences, Nanning, Guangxi China; 2 School of Clinical Medicine, Guilin Medical University, Guilin, Guangxi China; 3 Graduate school, Guangxi University of Chinese Medicine, Nanning, Guangxi China; 4 School of Clinical Medicine, Guangxi Medical University, Nanning, Guangxi China; 5 Center for Reproductive Medicine and Genetics, The People’s Hospital of Guangxi Zhuang Autonomous Region and Guangxi Academy of Medical Sciences, Nanning, Guangxi China

**Keywords:** Β-thalassemia, Isothermal amplification, Recombinase aided amplification (RAA), Reverse dot blot (RDB), Point-of-care testing (POCT)

## Abstract

Molecular diagnostic methods largely rely on expensive equipment and complex operations, which makes it difficult to achieve rapid and low-cost detection. In recent years, the increasing demand for point-of-care testing has driven the rapid development of isothermal amplification techniques, due to their simplicity and low equipment requirements. However, complex nucleic acid extraction steps are still required before most isothermal amplification. In this study, we propose a nucleic acid extraction-free gene detection method: direct multiplex Recombinase Aided Amplification combined with reverse dot blot hybridization (dmRAA-RDB). This method can detect multiple targets simultaneously and offers advantages such as high sensitivity, high specificity, low cost, no need for expensive instruments, and visual detection. By pre-treating whole blood samples with sodium hydroxide solution, the samples can be directly used for isothermal amplification and combined with commercial reverse dot blot (RDB) technology to rapidly detect 17 types of β-thalassemia mutations. The experimental results demonstrated that the pre-treated whole blood samples allowed for the simultaneous, stable, and efficient enrichment of all three target fragments via direct multiplex isothermal amplification. This single-tube triple amplification strategy demonstrates significant innovation in the field of isothermal amplification. Moreover, the workflow is shortened by nearly half, and a side-by-side comparison of 60 clinical samples showed 100 % agreement with the commercial PCR-RDB kit. The dmRAA-RDB method offers a highly promising, innovative solution for large-scale, rapid, and low-cost β -thalassemia screening in primary-care and resource-limited settings, and opens a new avenue for detecting other mutant genes.

**Supplementary Information:**

The online version contains supplementary material available at 10.1007/s00277-025-06711-5.

## Introduction

Thalassemia is a hereditary blood disorder, primarily caused by mutations in the globin genes that lead to an imbalance in the synthesis of globin chains, thereby causing hemolytic anemia [1]. Based on the type of gene mutation, the disease can be divided into two major categories: α-thalassemia and β-thalassemia. Normal adult hemoglobin is composed of α₂β₂ globin chains. When there is an abnormality or deletion in the β-globin chain gene, it leads to the occurrence of β-thalassemia [2].Currently, the primary cause of β-thalassemia is point mutations in the β-globin gene, and in a few cases, it is caused by base deletions or insertions [3].Globally, over 300 mutations related to β-thalassemia have been identified, and in the Chinese population, more than 129 point mutations in the β-globin gene have been discovered, among which over 90% are caused by 40 common mutations [4]. In the southern regions of China, the mutation types of β-thalassemia have certain regional characteristics and the more common mutations include CD41-42 (-CTTT), −28 (A >G), CD17 (AAG >TAG), CD26 (GAG >AAG), IVS-II-654 (C >T), and CD71-72 (+ A) [4, 5].These six mutations account for over 90% of all mutation types and represent the primary genetic background of β-thalassemia in the region. Patients with homozygous mutations (carrying two identical mutant genes) or compound heterozygous mutations (carrying two different mutant genes) typically present with severe anemia and jaundice [6]. This disease not only severely affects the health of the patients themselves but also brings significant economic and psychological pressures to families and society. Therefore, early diagnosis and intervention are of great significance for improving patient prognosis and reducing the disease burden.

Nowadays, molecular diagnostic techniques have been widely applied in disease diagnosis. Among them, PCR and qPCR technologies have become mainstream due to their high accuracy and sensitivity [7, 8]. However, their reliance on expensive equipment and specialized laboratories limits the demand for rapid and low-cost testing. In recent years, with the increasing demand for Point-of-Care Testing (POCT), isothermal amplification techniques such as Recombinase Polymerase Amplification (RPA), Loop-Mediated Isothermal Amplification (LAMP), Rolling Circle Amplification (RCA), and Recombinase Aided Amplification (RAA) have developed rapidly because of their ease of operation, low equipment requirements, rapid reaction, and high sensitivity [9, 10].

At present, the genetic diagnosis of thalassemia generally adopts a hierarchical strategy: first, preliminary screening is conducted via routine blood tests and hemoglobin electrophoresis; subsequently, Gap-PCR and RDB/ARMS-PCR are used to detect common deletion types and point mutations, respectively; finally, Sanger sequencing or next-generation sequencing (NGS) is employed as a supplementary method for difficult cases to identify any missed abnormalities [11, 12]. Although this system is mature and covers a comprehensive range of detection targets, its core workflow still heavily relies on professional laboratories, sophisticated instruments, and well-trained technicians. This leads to a long detection cycle and high costs, making it difficult to popularize in primary medical institutions and large-scale on-site screening. Therefore, there is an urgent need to develop a new complementary point-of-care testing (POCT) solution that is rapid, simple, and low-cost.

Reverse Dot Blot (RDB) is a high-throughput and easy-to-operate molecular detection technology. It allows simultaneous detection of multiple specific target sequences (such as pathogens or gene mutations) on a single membrane strip, with the advantages of low cost and intuitive results [13, 14]. However, its sensitivity and the target sequences to be detected are fixed during the design process, making it impossible to flexibly add new targets or achieve accurate quantification. Therefore, RDB is suitable for rapid screening of known targets, but not for scenarios that require the discovery of unknown targets or quantitative analysis.

Recombinase Aided Amplification (RAA) is a rapid and convenient nucleic acid amplification technique [15, 16]. The principle of this technology is based on the formation of a complex between recombinase and primers under isothermal conditions, which subsequently binds to the complementary sequences of the template DNA. With the assistance of single-stranded DNA-binding protein, the double-stranded structure of the template DNA is unwound, and DNA polymerase then synthesizes new DNA strands, thereby achieving exponential amplification of nucleic acids. Under an isothermal environment of 37–42 °C, the amplification process can be completed in just 10–30 min. This method does not rely on conventional PCR instruments and can be achieved using only a constant-temperature water bath, making it an ideal method for on-site rapid detection [17]. However, this technology (RAA) is currently only capable of amplifying DNA or RNA templates, necessitating a strict nucleic acid extraction step prior to amplification to obtain the required nucleic acid templates [18]. This limitation not only increases the complexity of the detection process but also, to some extent, restricts the further application of this technology in the field of point-of-care testing (POCT). In contrast, other isothermal amplification technologies such as LAMP and RPA have been innovatively improved by researchers to achieve direct amplification without nucleic acid extraction. For instance, Xiaonan Liu et al. developed a method called Direct-LAMP for rapid SNP genotyping; Mi-Ju Kim et al. utilized direct LAMP to identify subspecies of Enterobacteriaceae in probiotic products; and Goro Choi et al. developed a direct RPA microdevice for the real-time detection of foodborne pathogens in milk samples [19–21]. These advancements provide valuable references for the development of nucleic acid extraction-free RAA detection methods.

Despite the widespread application of recombinase-mediated isothermal amplification techniques (such as RAA/RPA) in pathogen detection due to their rapidity and efficiency, most current research remains confined to the development of single-target detection systems [22]. In recent years, multiplex detection technologies have gradually advanced, with some strategies adopting a “single-tube, single-target” microfluidic parallel detection approach [23]. While this enables multi-target detection to some extent, it still faces significant limitations in terms of operational convenience and detection throughput. Although several studies have successfully established single-tube duplex or triple real-time RT-RAA methods, thereby validating the feasibility of multiplex RAA technology, achieving efficient and synchronous amplification of three or more targets in a single reaction system while maintaining high sensitivity and specificity remains a technical challenge and a research frontier in the field [24, 25]. In this context, this study developed a single-tube triple RAA assay capable of simultaneously amplifying three segments of the β-thalassemia gene and detecting 17 associated mutations in one step. Through systematic optimization in primer/probe design and reaction conditions, the proposed method achieves robust co-amplification of three targets, further advancing the potential application of multiplex RAA technology in rapid genetic disease screening. In addition, the method developed in this study is also a nucleic acid extraction-free, rapid, and low-cost detection method: by subjecting whole blood samples to a simple 3-minute lysis treatment with sodium hydroxide solution, the samples can be directly used as templates for Recombinase Aided Amplification (RAA) reactions. By combining this isothermal amplification technique with Reverse Dot Blot (RDB) technology, we can achieve specific amplification and visual detection of β-thalassemia-related mutation sites under rudimentary laboratory conditions. This approach establishes a streamlined and low-cost process from sample to result, significantly reducing the reliance on specialized laboratories and cutting the detection time by more than half. This innovative approach, hence named dmRAA-RDB, offers a groundbreaking solution for rapid and cost-effective screening of hereditary blood diseases.

## Materials and methods

### Sample collection

In this study, we collected peripheral blood samples from 50 patients who were diagnosed with β-thalassemia through genetic testing, as well as from 10 patients who were excluded for β-thalassemia through genetic testing. Each sample was 2 mL in volume and anticoagulated with EDTA-K2, and then stored in a −80℃ freezer. All samples were subjected to both PCR Reverse Dot Blot (PCR-RDB, provided by Yane Biotech) and dmRAA-RDB assays. The study was approved by the Ethics Committee of our hospital, and informed consent was obtained from all patients.

### Instruments and reagents

Recombinase Aided Amplification (RAA) kit (Hangzhou Zhongce Biotechnology Co., Ltd., China), β-thalassemia PCR-Reverse Dot Blot (PCR-RDB) Detection Kit (Yane Biotechnology (Shenzhen) Co., Ltd., China), constant temperature water bath (Changzhou Yinan Laboratory Instrument Factory, China), PCR-RDB reagents and fully automated nucleic acid hybridization instrument (Yane Biotechnology (Shenzhen) Co., Ltd., China).

### Primer design

Primers were designed in accordance with the principles of RAA primer design (primer length: 30–35 bp; amplicon length not exceeding: 500 bp). A total of three pairs of specific primers were designed for RAA amplification, with biotin labeling at the 5′ end of each primer. The target amplicon lengths were 329 bp, 380 bp, and 441 bp, respectively, covering all the mutation sites to be detected in this study of β-thalassemia. The primer sequences are detailed in Table S1(supplementary materials).

### Establishment and optimization of DmRAA amplification system

The RAA amplification reagent used in this study was a basic RAA kit purchased from Hangzhou Zhongce Bio-Sci & Tech Co., Ltd, China. To establish the optimal dmRAA amplification system, we systematically evaluated key conditions, including the sodium hydroxide pretreatment ratio of the sample and primer concentration. Experimental results indicated that the dmRAA amplification system performed relatively stably when whole blood was pretreated with 0.1 M sodium hydroxide solution at a 1:4 ratio as the amplification template, the primer concentration was 10 µM, and the reaction was carried out at 39 °C for 30 min.

**Fig. 1:**
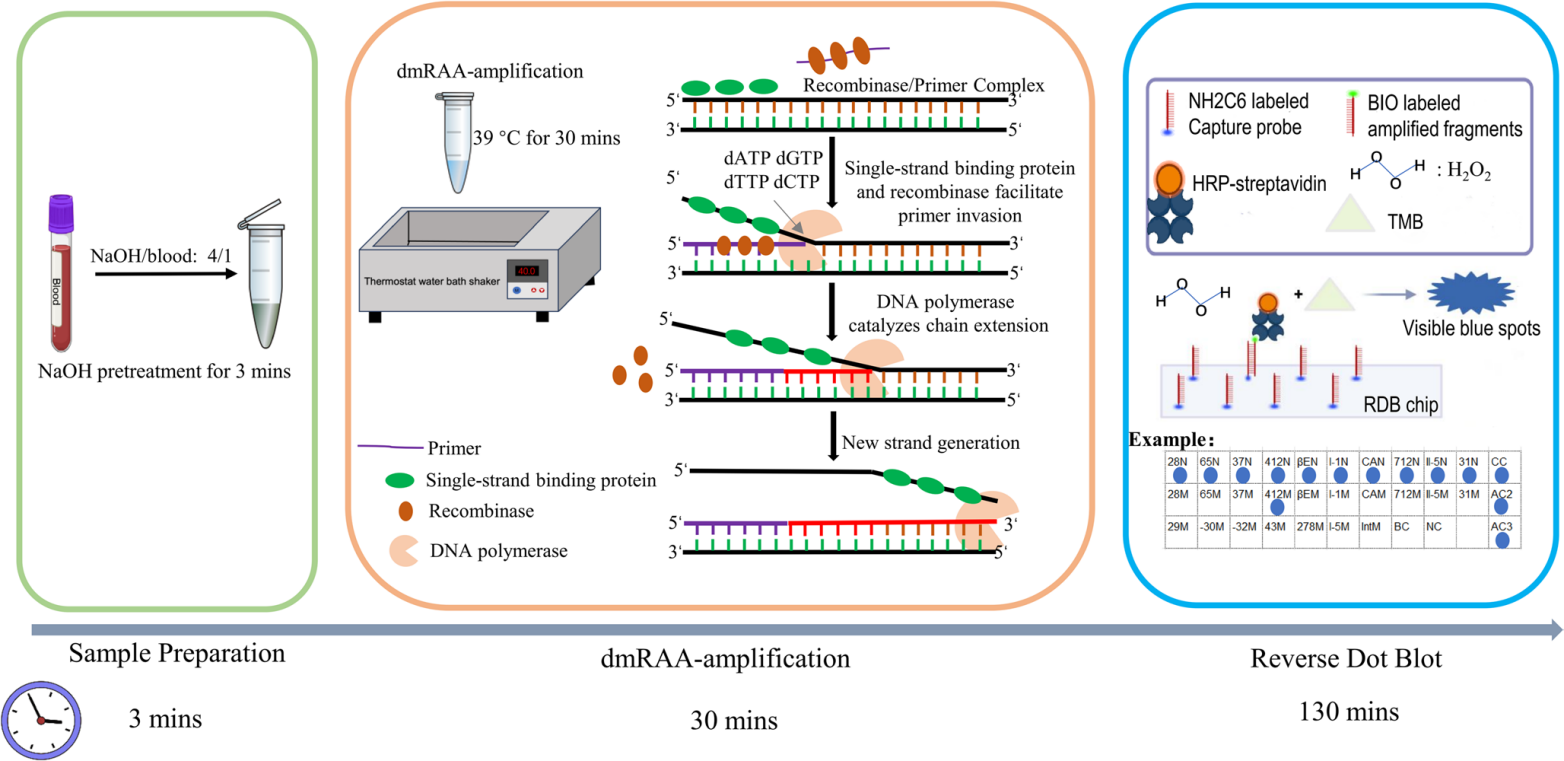
Schematic diagram of the principle of the dmRAA-RDB method and the layout of the hybridization membrane

The total volume of each amplification reaction was 50 µL, and the reaction system was prepared as follows: First, 25 µL of Buffer A, an appropriate amount of the 10 µM forward and reverse primer mixture, and supplementary ddH₂O were added to the reaction tube provided in the RAA kit. Then, 5 µL of the alkali-treated template (whole blood pretreated with 0.1 M NaOH at a 1:4 ratio) was added, followed by Buffer B containing recombinase. The reaction mixture was thoroughly mixed and subjected to low-speed centrifugation. The detection unit tubes were then incubated in a constant-temperature metal bath or water bath for the amplification reaction. The principle of RAA amplification is shown in Fig. 1.

### Probe design for RDB

The human HBB gene (i.e., the “β-globin gene”) genomic sequence is from the GenBank database. Based on the mutation information of β-thalassemia to be detected, specific probes were designed using SnapGene software (GSL Biotech, USA). We selected 17 common β-thalassemia gene mutation types in China and designed a total of 31 specific capture probes, including 10 probes for detecting wild-type sites (indicated by “N”), 17 probes for detecting mutant sites (indicated by “M”), 2 positive control probes (AC2, AC3), 1 negative control probe (NC), and 1 colorimetric system monitoring probe (CC). The blank control site is BC. The 5′ end of the CC probe is labeled with NH2 C6, and the 3′ end is labeled with biotin; the remaining probes are labeled with NH2 C6 at the 5′ end and are unlabeled at the 3′ end. The probe sequences are detailed in Table S1(supplementary materials). The correspondence of probes and HGVS nomenclature are detailed in Table S2(supplementary materials).

Result interpretation: If the probe “M” for a mutation site shows color, it indicates that the patient carries the mutant genotype. If the corresponding wild-type probe “N” does not show color, it is a homozygous mutation; if the probe “N” shows color, it is a heterozygous mutation. In addition, if the CC probe site does not show color, it indicates that there was an error in the hybridization step. When the CC probe shows color but all other probe sites do not show color, it indicates an error in the RAA amplification system. If the positive control probes AC2 and AC3 do not show color, or if the NC and BC sites show color, the current results are unreliable and need to be retested.

### Preparation of RDB membrane

The membrane used for chip preparation is a nylon transfer membrane (Biodyme C (0.45 μm)) purchased from PALL Company. The process for preparing the gene chip membrane is as follows: (1) Print the chip membrane layout using a black and white laser printer; (2) Prepare the probe working solution (0.1 µM); (3) Activate the chip membrane with a 10% EDAC solution; (4) Immobilize the probes: Spot the probes onto the nylon membrane, 0.5 µl per well; (5) Terminate the reaction by soaking the chip membrane in a 0.1 M NaOH solution, resulting in the prepared chip membrane. The detailed information of the chip membrane layout is shown in the example in Fig. 1.

### RDB detection system

The reverse dot blot hybridization experiment can be completed using the fully automatic nucleic acid hybridization instrument produced by Yane Bio Co., Ltd. When conditions are limited, it can also be manually performed using only a constant temperature water bath. The hybridization reaction buffer (20× SSC, 10% SDS, 1 M sodium citrate buffer) was purchased from Yane Bio Co., Ltd. Streptavidin-HRP and 3,3’,5,5’-tetramethylbenzidine (TMB) were purchased from APE BioReagents, USA. The final determined conditions for reverse dot blot hybridization are as follows: the amplified products are hybridized at 50 °C for 60 min, HRP incubated for 25 min, and TMB color development for 10 min. The principle of the dmRAA-RDB method is detailed in Fig. 1.

## Results

### Screening of DmRAA primers and optimization of the reaction system

Based on the fundamental requirements of RAA technology for primer design (primer length: 30–35 bp, amplification product length not exceeding 500 bp, GC content: 40%–60%), this study designed and screened multiple pairs of primers. To improve detection efficiency, we attempted to simultaneously amplify multiple target fragments in a single-tube reaction system. Through systematic screening and validation, three pairs of specific primers were ultimately selected, capable of amplifying DNA fragments with lengths of 329 bp, 380 bp, and 441 bp, respectively, achieving multiplex amplification.

To determine the optimal reaction conditions, we compared the amplification effects under four primer concentrations: 1 µM, 5 µM, 10 µM, and 20 µM. Gel electrophoresis results showed that the amplification bands at a primer concentration of 10 µM were clear and bright, with no significant difference compared to the results under 20 µM conditions, while also exhibiting higher reagent usage efficiency. In contrast, the band signals under 1 µM and 5 µM conditions were weaker. Therefore, 10 µM was determined as the optimal primer concentration.

**Fig. 2:**
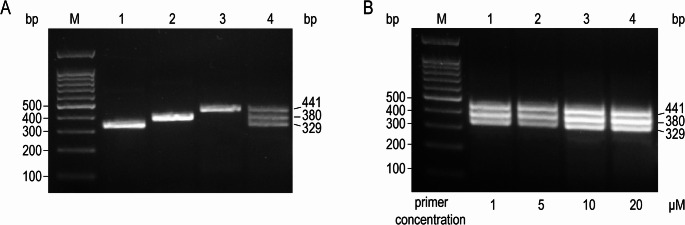
dmRAA amplification of β-thalassemia gene fragments. A: Lane M: Marker. Lane 1: Primer pair 1 amplifies a 329 bp fragment. Lane 2: Primer pair 2 amplifies a 380 bp fragment. Lane 3: Primer pair 3 amplifies a 441 bp fragment. Lane 4: All three primer pairs amplify 329 bp, 380 bp, and 441 bp fragments in a single-tube reaction. B: Exploration of the optimal primer concentration

The final established dmRAA reaction system included 5 µL of preprocessed sample template and three pairs of primers (2 µL each, at a concentration of 10 µM). Amplification was completed under constant temperature conditions at 39 °C for 30 min. The results of single-tube multiplex amplification and primer concentration optimization validation are shown in Fig. 2A and B, respectively. All experiments were independently repeated three times to ensure result reliability.

### Determination of the probes

In reverse dot blot (RDB)-based β-thalassemia testing, probe selection is a critical step to ensure detection accuracy. Typically, for each common mutation, a pair of oligonucleotide probes fully complementary to the wild-type and mutant sequences, respectively, is designed to effectively distinguish between homozygous and heterozygous genotypes. In this experiment, a total of 31 probes were used, including 10 wild-type probes (labeled “N”), 17 mutant probes (labeled “M”), two positive controls (AC2, AC3), one negative control (NC), and one chromogenic system control probe (CC). The number of wild-type probes exceeds that of mutant probes because some adjacent mutation sites can share the same wild-type probe. The probe design follows these principles: a length of approximately 20 bp, the mutation site positioned centrally, and similar Tm values across all probes. Subsequently, hybridization experiments were conducted using samples of known genotypes validated by sequencing or other reference methods. By systematically optimizing hybridization conditions (such as temperature and time), probes capable of clearly distinguishing between perfectly matched and mismatched hybridization were selected. For probes that could not be effectively differentiated through condition optimization, their sequences were redesigned, with common strategies including adjusting probe length or GC content. The ultimate goal is to obtain a probe combination with high specificity and accuracy, ensuring that wild-type samples only bind to wild-type probes, while mutant samples correctly bind to the corresponding mutant probes. The probe sequences are detailed in Table S1(supplementary materials).

### Optimization of DmRAA sample pre-treatment

Although RAA isothermal amplification techniques do not require expensive amplification instruments and can significantly shorten the amplification cycle, their amplification templates are limited to nucleic acids. Therefore, a strict nucleic acid extraction step must be performed before amplification to obtain the DNA or RNA template for amplification. This limitation not only increases the complexity of the detection process but also restricts the further application of this technology in point-of-care testing (POCT). To overcome this issue, we attempted to directly perform RAA isothermal amplification on whole blood samples after simple pre-treatment.

**Fig. 3:**
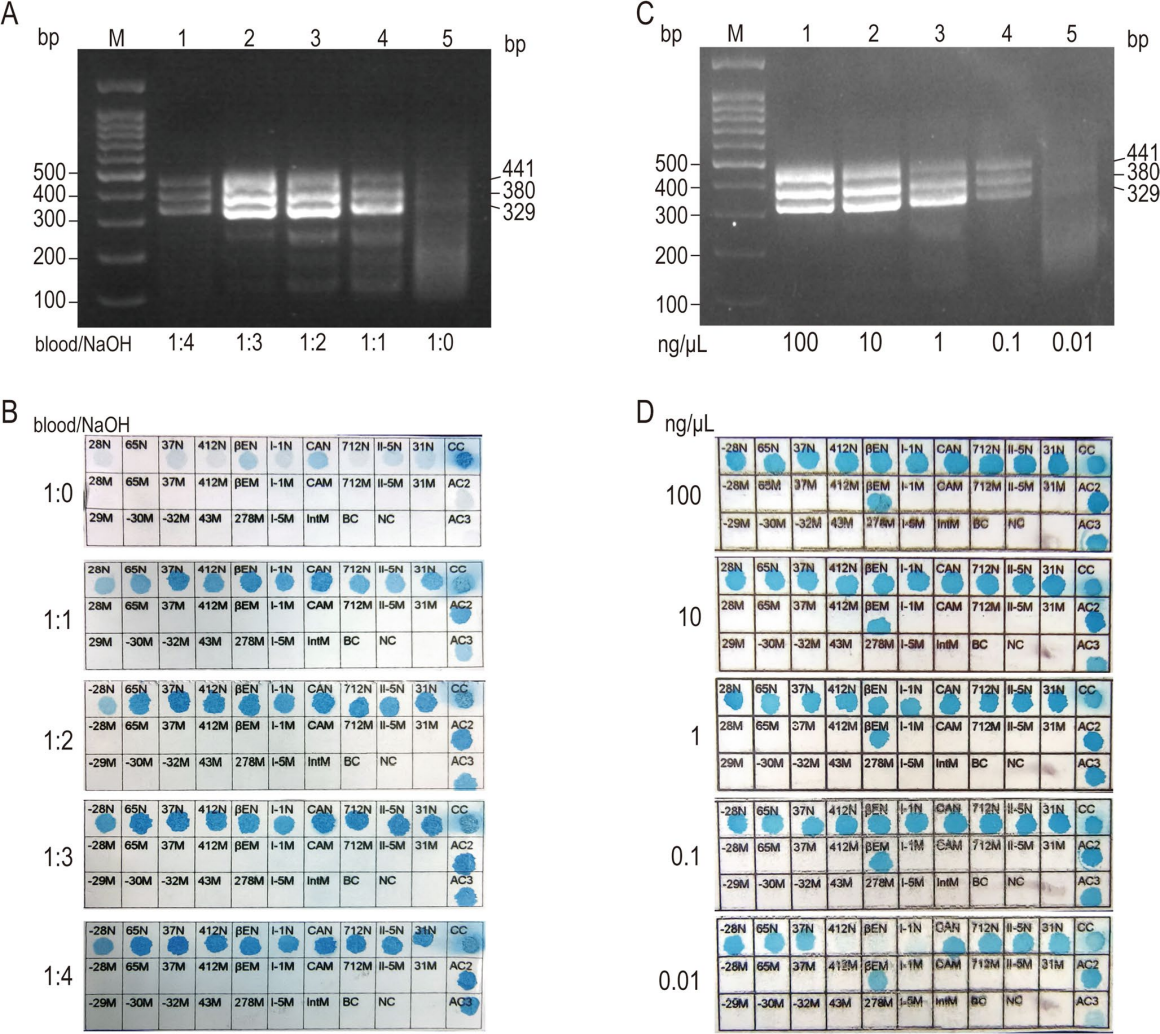
Exploration of the optimal sodium hydroxide pre-treatment ratio and detection sensitivity. A: Lane M is the Marker. Lanes 1–5 show the direct multiplex RAA amplification results of whole blood/sodium hydroxide (1:4, 1:3, 1:2, 1:1, 1:0) mixtures, respectively. B: Hybridization detection results of samples pre-treated with different ratios. C: Lane M is the Marker. Lanes 1–5 show the electrophoresis results of dmRAA amplification of DNA samples with concentrations of 100, 10, 1, 0.1, and 0.01 ng/μL, respectively. D: Hybridization detection results of samples with different DNA concentrations

In the experiments, we tested different ratios of whole blood to sodium hydroxide solution (0.1 M) (1:0, 1:1, 1:2, 1:3, 1:4) to evaluate their effects on amplification and hybridization. Analysis of the results from agarose gel electrophoresis and reverse dot blot hybridization showed that the optimal amplification and hybridization effects were achieved when the ratio of whole blood to sodium hydroxide solution was 1:4 (Fig. 3A and B). All experiments were repeated three times.

### Detection sensitivity

Since the pre-treated samples obtained by mixing whole blood with sodium hydroxide still contain blood components and cannot be directly measured for DNA concentration, we used purified DNA templates instead of pre-treated samples to explore the detection sensitivity of multiplex RAA.

We randomly selected blood samples from three patients with known genotypes of β-thalassemia, extracted nucleic acids from these samples, and measured the DNA concentration using a NanoDrop spectrophotometer. Subsequently, the DNA samples were diluted to a gradient of concentrations: 100 ng/µL, 10 ng/µL, 1 ng/µL, 0.1 ng/µL, and 0.01 ng/µL using nuclease-free water. For each gradient concentration of DNA, we performed dmRAA amplification and reverse dot blot hybridization. Additionally, agarose gel electrophoresis was conducted on the multiplex RAA amplification products to further verify the amplification results. The experimental results showed that the detection sensitivity of this method could reach 0.1 ng/µL. The agarose gel electrophoresis results showed no effective target fragments at the sample concentration of 0.01 ng/µL, and the reverse dot blot hybridization results were invalid (Fig. 3C and D).

### Specificity (anti-interference capability)

**Fig. 4:**
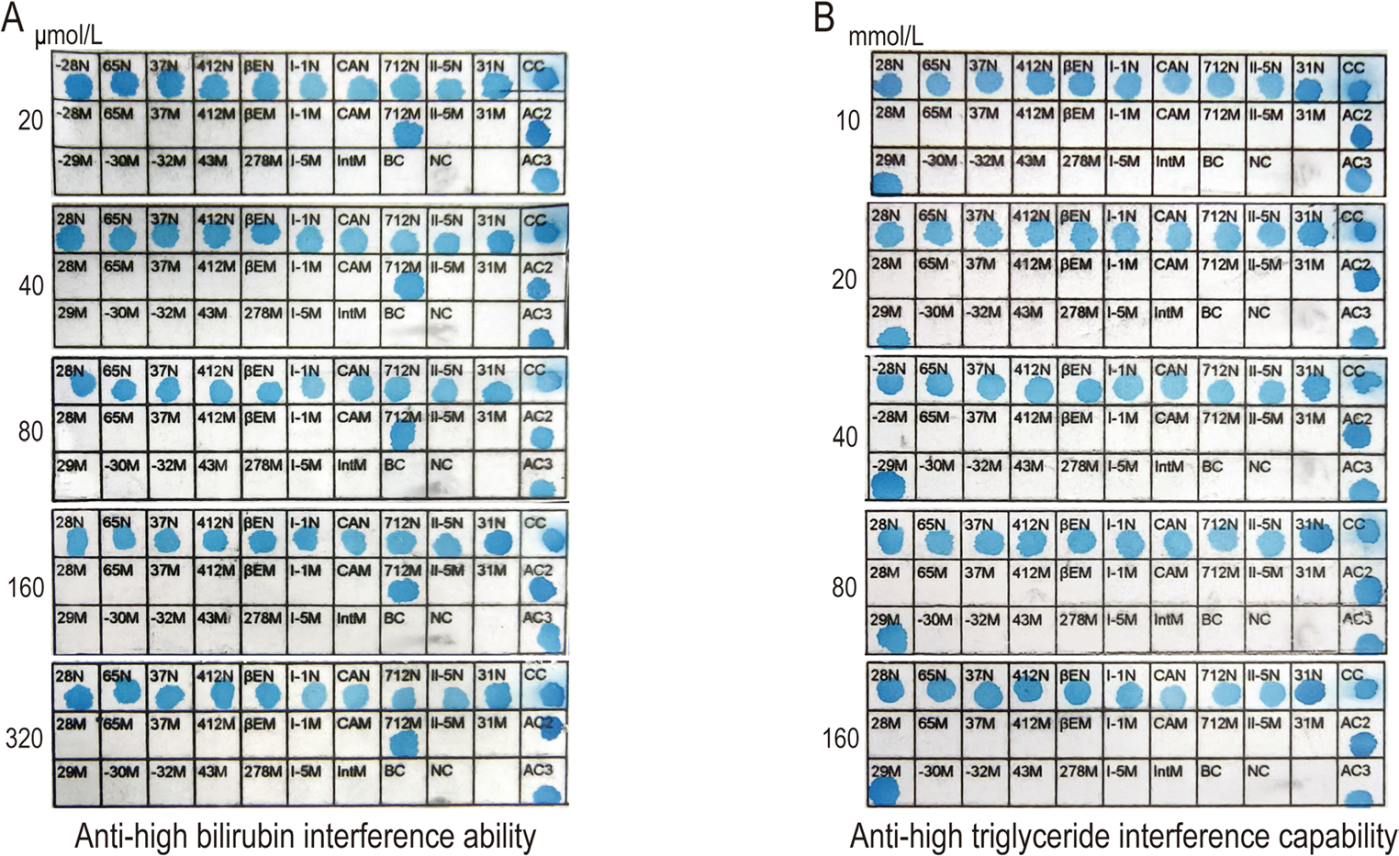
Anti-interference capability. A: High bilirubin samples: Results of dmRAA-RDB detection for samples with bilirubin concentrations of 20 μmol/L, 40 μmol/L, 80 μmol/L, 160 μmol/L, and 320 μmol/L. B: High triglyceride samples: results of dmRAA-RDB detection for samples with triglyceride concentrations of 10 mmol/L, 20 mmol/L, 40 mmol/L, 80 mmol/L, and 160 mmol/L

Since the amplification samples used in the experiment were simply pre-treated whole blood samples, common blood components such as bilirubin and triglycerides may interfere with the detection results when their concentrations are elevated. To evaluate the applicability and stability of this method in complex biological samples, we further assessed its anti-interference capability against high concentrations of bilirubin and triglycerides. We prepared blood samples with different concentrations of bilirubin and triglycerides, as follows: High bilirubin samples: 20 µmol/L, 40 µmol/L, 80 µmol/L, 160 µmol/L, and 320 µmol/L. High triglyceride samples: 10 mmol/L, 20 mmol/L, 40 mmol/L, 80 mmol/L, and 160 mmol/L. These samples were subjected to dmRAA amplification and reverse dot blot hybridization detection. The results showed that even with bilirubin concentrations as high as 320 µmol/L or triglyceride concentrations as high as 160 mmol/L, the method was still able to accurately detect the mutation types. This indicates that the dmRAA detection method has good anti-interference capability in the presence of high concentrations of bilirubin and triglycerides and can be applied to the detection of complex blood samples. The detection results are shown in Fig. 4. All experiments were repeated three times.

Furthermore, under the standardized sample collection conditions adopted in this study (i.e., uniform use of EDTA-K₂ vacuum blood collection tubes compliant with the Chinese medical industry standard YY 0314–2007, with an anticoagulant content of 1.5–2.2 mg/mL of blood), no significant impact of common anticoagulants (such as EDTA) or sample hemolysis on subsequent amplification and hybridization results was observed. Additionally, the detection method demonstrated no cross-reactivity with α-thalassemia or β-thalassemia genotypes located outside the chip membrane region.

### Clinical application and methodological comparison

To verify the capability of the dmRAA-RDB method for detecting β-thalassemia, we selected 60 clinical samples whose genotypes had been determined by the PCR-RDB technique (using the β-thalassemia detection kit from Yaneng). We conducted a single-blind trial using the β-thalassemia dmRAA-RDB method. The negative and positive concordance rates between the two methods were both 100%, with a kappa value of 1 (Table 1).

**Table 1:** Method conformity verification

		Reference Method (PCR - RDB)		Total Number
		Positive	Negative	
(dmPCR – RDB)	Positive	50	0	50
	Negative	0	10	10
Total Number		50	10	60

Note:The reference method is PCR - RDB method, and the β - thalassemia detection kit is produced by yaneng company

Positive Agreement Rate = 83/(83 + 0)*100%=100%; Negative Agreement Rate = 66/(66 + 0)*100%=100%. KAPPA = 1. Positive indicates that the detected mutation type is consistent, while negative indicates that no mutation is detected

**Fig. 5:**
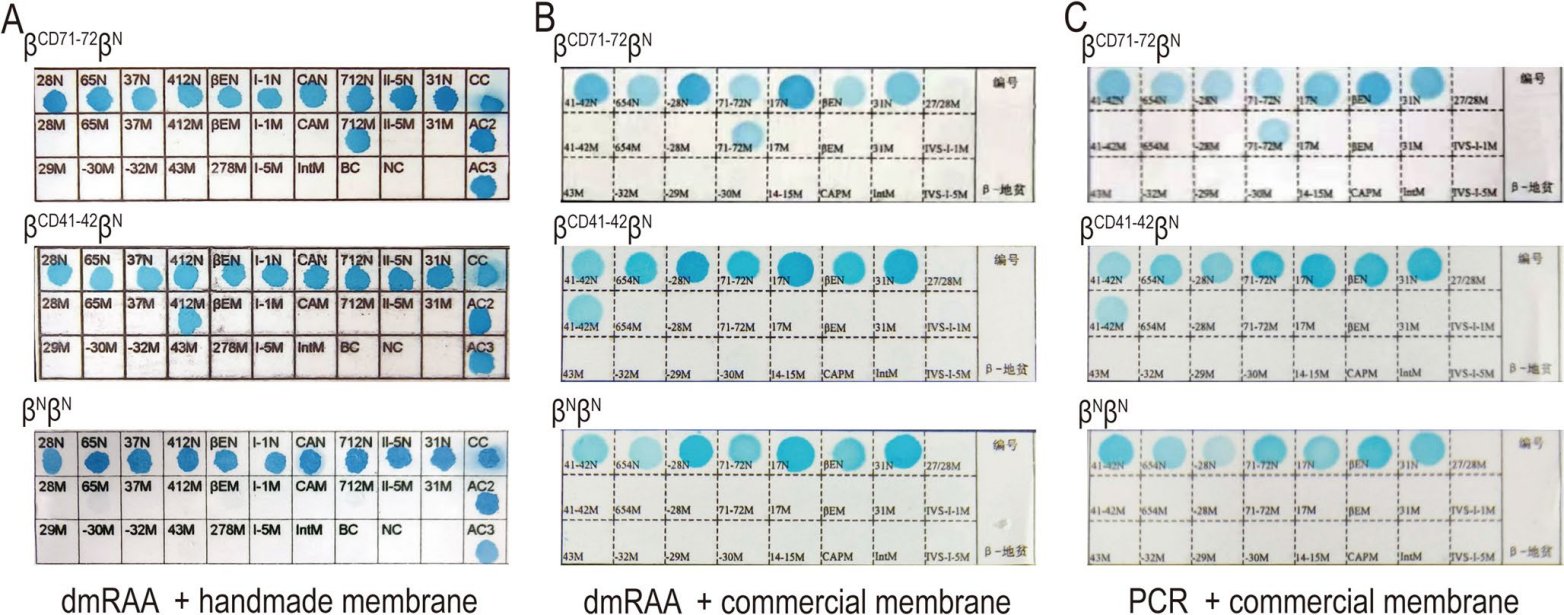
Methodological comparison. A: The amplified products of dmRAA were subjected to reverse dot blot hybridization with manual membranes. B: Hybridization reaction of dmRAA amplification products with commercial reverse dot blot hybridization membrane. C: Hybridization reaction results of conventional PCR amplification products with commercial reverse dot blot hybridization membrane

In addition, we performed cross-hybridization reactions between the dmRAA amplification products and the commercial reverse dot blot hybridization membrane from Yaneng Company to assess the universality of the dmRAA amplification method. Moreover, by comparing the detection results with those of the commercial detection kit, we further validated the performance of this method in terms of result visualization and the convenience of the testing process. Some of the comparative results are shown in Fig. 5.

## Discussion

Thalassemia is one of the most common autosomal recessive genetic disorders worldwide, primarily prevalent in tropical and subtropical regions [26, 27]. The disease is caused by a variety of complex genetic mutations, and its clinical manifestations vary significantly depending on genetic background, mutation type, and environmental factors, ranging from asymptomatic carriers to severe hemolytic disease in newborns [28]. Nevertheless, thalassemia is still a genetic disorder that can be effectively prevented through scientific means [29]. By establishing rapid and efficient diagnostic methods, it is possible to achieve widespread screening for thalassemia gene mutations, thereby enabling early detection, early intervention, and precise treatment [30]. This not only significantly reduces the birth rate of children with thalassemia but also effectively alleviates the medical burden on families and society, providing important support for public health [31].

Currently, nucleic acid amplification-based genetic testing technologies have demonstrated broad application potential in the diagnosis of human genetic diseases, detection of drug-resistant genes, and screening of pathogens [10, 32]. As a fundamental step in genetic diagnostics, traditional PCR amplification technology, with its high sensitivity and specificity, has been widely used in large laboratories and specialized testing institutions [33]. However, PCR technology relies on complex thermal cycling equipment, which poses many limitations for its application in low-resource settings. In contrast, isothermal amplification techniques, which do not require thermal cycling, have a shorter amplification cycle and lower equipment requirements, are gradually becoming the focus of researchers [34, 35]. In recent years, significant breakthroughs have been made in the key performance indicators of isothermal amplification technology. By integrating with the CRISPR-Cas system, the detection sensitivity of technologies such as RPA, RAA and LAMP have been enhanced to the single-copy level, while their specificity has significantly improved, effectively suppressing non-specific amplification [36].The introduction of microfluidic and lyophilization technologies has further reduced the reaction time to 10–20 min and supports transportation and storage at room temperature, providing robust support for point-of-care testing (POCT). In the field of clinical diagnostics, these technologies have been widely applied for the detection of infectious disease pathogens, including the HBV, hand-foot-and-mouth disease, and HIV [37–42]. Some products have already received Class Ⅲ certification from the National Medical Products Administration (NMPA), enabling parallel screening of over 20 respiratory pathogens within 1.5 h, with sensitivity reaching the pg/mL level. Additionally, the combined application of nanopore sequencing and LAMP technology allows for rapid identification and tracking of pathogens within one hour, further expanding the potential of this technology in genetic disease diagnosis and early cancer screening [43].

In this context, recombinase-aided isothermal amplification (RAA), selected as a representative technology in this proof-of-concept study, demonstrated its applicability under specific experimental conditions: this method requires only a constant temperature of 39 ℃ to complete amplification, featuring short reaction times and relatively straightforward primer design. Moreover, during the course of this study, the relevant reagents were readily accessible, and the overall cost was low, as amplification could be achieved using basic constant-temperature equipment such as a water bath, thereby offering technical feasibility for genetic testing in resource-limited settings [44]. However, RAA still has certain limitations. For instance, its templates are limited to DNA or RNA, and in actual detection processes, it generally still relies on stringent nucleic acid extraction steps to obtain effective templates, which complicates the overall procedure and has not yet fully eliminated the dependence on extraction equipment. Therefore, future research should focus on further optimizing RAA-based detection workflows to reduce their reliance on equipment and operational conditions. Meanwhile, the integrated “isothermal amplification–reverse dot blot hybridization” strategy proposed in this study, as a versatile technical framework, also holds potential for adaptation to other isothermal amplification methods such as RPA and LAMP, thereby promoting the development of more flexible and multiplex nucleic acid detection systems.

In the field of bacterial detection and related areas, direct detection strategies based on chemical lysis or heat treatment have been successfully applied to various isothermal amplification techniques such as RPA and LAMP [19, 20]. Furthermore, clinical practice has validated that simple pretreatment with sodium hydroxide enables direct LAMP amplification of samples such as whole blood, dried blood spots, oral swabs, and saliva. This method, referred to as Direct-LAMP, has achieved accurate genotyping of single nucleotide polymorphisms (SNPs) [21]. Based on these advancements, this study proposes a detection method that eliminates the need for traditional nucleic acid extraction steps: whole blood samples pretreated with sodium hydroxide solution can be directly used for recombinase-aided amplification (RAA) to efficiently amplify target fragments in the genome. The core of this method lies in utilizing the chemical properties of NaOH solution to rapidly disrupt the cell membranes and nuclear structures of cells in whole blood, thereby releasing the genomic DNA from nucleated cells [21]. At the same time, NaOH solution can effectively inactivate the natural DNA polymerase inhibitors present in whole blood, including hemoglobin, IgG, lactoferrin, and proteases, thereby eliminating the potential interference of these substances on the amplification reaction [45]. By pre-treating whole blood samples with NaOH solution at a specific ratio, the detection process can be significantly simplified, eliminating the need for complex equipment and time-consuming operations required for traditional nucleic acid extraction. The treated whole blood samples can be directly used as templates for RAA isothermal amplification without further purification, thus completing the gene amplification. This approach provides an efficient and low-cost solution for gene detection in resource-limited settings.

The Reverse Dot Blot (RDB) technique was first introduced by Saiki et al. in 1989. This technology is second only to DNA sequencing in terms of accuracy for detecting gene mutations and can detect multiple point mutations in a single experiment [13, 46]. This technology has been widely applied in the detection of various pathogens, including human papillomavirus (HPV) genotyping, hepatitis B virus (HBV) genotyping and drug resistance testing, as well as non-tuberculous mycobacteria (NTM) identification [47–49]. Compared with fluorescent PCR technology, the RDB technique can cover a greater number of targets in a single detection, thereby achieving higher throughput. Moreover, fluorescent PCR technology is limited by its dependence on expensive equipment, whereas the hybridization process of the RDB technique can be completed with a simple constant-temperature water bath shaker. Additionally, the RDB technique visualizes detection results through an enzymatic colorimetric reaction, making it particularly suitable for grassroots laboratories with limited medical equipment resources.

Based on this, we combined direct multiplex Recombinase Aided Amplification (dmRAA) with the Reverse Dot Blot (RDB) method to develop a rapid detection method for β-thalassemia that does not require expensive equipment. This combination also provides a novel approach for the detection of other mutant genes. Our research results indicate that after sample pretreatment with NaOH solution and whole blood at a ratio of 1:4 for 3 min, the amplification effect of dmRAA and the hybridization effect of RDB are optimal. Using three pairs of specific primers, three target fragments with lengths of 329 bp, 380 bp, and 441 bp, respectively, can be successfully amplified in a single tube. The sensitivity experiment results show that the detection sensitivity of this method can reach 0.1 ng/µL, demonstrating extremely high detection sensitivity. Moreover, high concentrations of bilirubin and triglycerides in whole blood samples do not affect the efficiency of dmRAA amplification and hybridization detection after simple pretreatment, indicating extremely high anti-interference ability. Finally, we compared our method with a commercial β-thalassemia detection kit using 60 known mutant β-thalassemia patient samples. The results showed that the consistency rate between the two methods could reach 100%. Compared to the commercial YaNeng kit that requires DNA extraction and hours-long PCR amplification, our method streamlines the entire process by reducing sample preparation to a one-step rapid lysis and shortening the amplification step to just 30 min, substantially improving testing efficiency (Table 2). Additionally, the commercial reverse dot blot membrane can still detect the mutant gene sites using the products amplified by dmRAA, indicating that the dmRAA amplification method has extremely high universality.

**Table 2:** Detection time comparison based on PCR-RDB methods

Testing Steps							
Method (Sample)	sample size	Sample Processing	PCR amplification	product denaturation	hybridization reaction	Result Analysis	Total time
Traditional PCR-RDB (DNA)	200 µl	1.5 h (Artificial extraction method)	2.5 h	15 min	2–3 h	10 min	≈6–7 h
dmRAA-RDB	5 µl	3 min	30 min	15 min	2–3 h	10 min	≈3–4 h

Note: The time taken by the Traditional PCR-RDB (DNA) method is referenced to the thalassemia detection kits sold by Yaneng Company.(China)

However, this study also has several limitations. First, our sample size was relatively small and primarily drawn from a Southern Chinese population, with the analyzed mutations also focusing on the common types in this region. Therefore, the performance of our method (e.g., sensitivity, specificity) for other ethnic groups, geographic regions, or rare mutation types still requires further validation in larger-scale and more diverse cohort studies. Furthermore, although this study preliminarily confirmed the feasibility of combining direct isothermal amplification with reverse dot blot hybridization for genetic disease detection, the current results still primarily rely on qualitative assessment and have not yet achieved systematic quantitative analysis of hybridization signals. This limitation somewhat restricts the precise definition of detection results in terms of sensitivity and specificity. In subsequent work, we will focus on developing an automated interpretation system compatible with smartphones or portable devices. By capturing the grayscale values of each probe spot on the membrane strip and integrating predefined thresholds, we aim to achieve rapid and objective “immediate visual interpretation.” Additionally, we will further optimize quantitative algorithms and advance the validation and application of this technology in more clinical samples to enhance its reliability and practicality as a screening tool. However, in terms of transitioning to POCT (Point-of-Care Testing), due to the technical principles of this method being based on isothermal amplification and reverse dot blot hybridization, it involves multiple operational steps, and the complete detection process typically takes several hours. As a result, under the constraints of factors such as detection duration and environmental requirements, transforming it into a truly practical POCT platform still faces significant challenges [41]. It is worth noting that in the practical application scenarios of genetic screening for human hereditary diseases, the core requirement for detection results is typically not extremely rapid feedback but rather high accuracy and reliability. Nevertheless, to advance this technology toward POCT, our subsequent research will focus on the following key aspects: First, it is necessary to integrate steps such as sample preprocessing, isothermal amplification, and hybridization detection into an integrated, fully sealed microfluidic chip or testing cartridge to achieve a simple “sample in, result out” operational model, thereby minimizing manual intervention and reducing the risk of cross-contamination [50].

In summary, in light of the existing detection methods’ shortcomings, such as cumbersome operations, reliance on expensive equipment, long duration, high costs, and inability to meet the clinical needs for rapid and low-cost testing, we have developed a novel method for β-thalassemia detection without nucleic acid extraction or expensive amplification instruments: the direct multiplex Recombinase Aided Amplification-Reverse Dot Blot (dmRAA-RDB) method. This method is expected to meet the gene detection needs for thalassemia in medical institutions at all levels and provide new ideas for the detection of other genes or mutations. With its high efficiency and convenience, the dmRAA-RDB method is likely to be widely applied in clinical laboratories in the future and play a significant role in the detection of thalassemia mutation genes.

## Supplementary Information

Below is the link to the electronic supplementary material.

ESM 1 (DOCX 15.1 KB)

ESM 2 (DOCX 16.2 KB)

Supplementary figure 6 (PNG 136 KB)

High Resolution Image (TIF 7.06 MB)

Supplementary figure 7 (PNG 149 KB)

High Resolution Image (TIF 7.11 MB)

Supplementary figure 8 (PNG 249 KB)

High Resolution Image (TIF 15.5 MB)

Supplementary figure 9 (PNG 285 KB)

High Resolution Image (TIF 15.5 MB)

ESM 7 (PNG 7.42 MB)

ESM 8 (PNG 5.66 MB)

ESM 9 (JPG 448 KB)

## Data Availability

No datasets were generated or analysed during the current study.
