# Supplementary material for: A direct multiplex isothermal amplification-reverse dot blot hybridization system for β-thalassemia diagnosis
Source: Ann Hematol. 2025 Nov 18;104(12):6147–59. doi: 10.1007/s00277-025-06711-5 (PMC12764633; doi:10.1007/s00277-025-06711-5)
Supplement: Supplementary file 1 — (DOCX 15.1 KB) [file 277_2025_6711_MOESM1_ESM.docx]

**Table S1: Probe and primer sequences**

| Sequence Number | Sequence Name | Sequence(5’to3‘) |
| --- | --- | --- |
| SEQ ID NO.1 | -28N | ACTTTTATGCCCAGCCCT |
| SEQ ID NO.2 | -32M | GGGCTGGGAATAAAAGTCAG |
| SEQ ID NO.3 | -30M | TGACTTTTGTGCCCAGCC |
| SEQ ID NO.4 | -29M | TGACTTTCATGCCCAGCC |
| SEQ ID NO.5 | -28M | CCCTGACTTCTATGCCCA |
| SEQ ID NO.6 | Cap+40-43N | CCATGGTGTCTGTTTGAGG |
| SEQ ID NO.7 | Cap+40-43M | AGCAACCTCAGACACCATG |
| SEQ ID NO.8 | Int M | CAGACACCAGGGTGCATC |
| SEQ ID NO.9 | CD26(βE)N | CCCAGGGCCTCACCAC |
| SEQ ID NO.10 | CD26(βE)M | GTTGGTGGTAAGGCCCTG |
| SEQ ID NO.11 | CD27-28M | GTGGTGAGGCCCCTGG |
| SEQ ID NO.12 | IVS-Ⅰ-1N | TGATACCAACCTGCCCAG |
| SEQ ID NO.13 | IVS-Ⅰ-1M | CCCTGGGCAGATTGGTATC |
| SEQ ID NO.14 | IVS-Ⅰ-5M | GGCAGGTTGCTATCAAGGTTA |
| SEQ ID NO.15 | CD31N | CCTTAGGCTGCTGGTGGT |
| SEQ ID NO.16 | CD31M | CCCTTAGGTGCTGGTGG |
| SEQ ID NO.17 | CD41-42N | ACCCAGAGGTTCTTTGAGTC |
| SEQ ID NO.18 | CD41-42M | ACCCAGAGGTTGAGTCCTTT |
| SEQ ID NO.19 | CD43M | AGAGGTTCTTTTAGTCCTTTGG |
| SEQ ID NO.20 | CD71-72N | GCTCGGTGCCTTTAGTGA |
| SEQ ID NO.21 | CD71-72M | TGCCTTTAAGTGATGGCCT |
| SEQ ID NO.22 | IVS-Ⅱ-654N | TTGCTATTGCCTTAACCCAG |
| SEQ ID NO.23 | IVS-Ⅱ-654M | TATTGCTATTACCTTAACCCAG |
| SEQ ID NO.24 | IVS-Ⅱ-5N | TCAGGGTGAGTCTATGGGA |
| SEQ ID NO.25 | IVS-Ⅱ-5M | TCCCATAGAGTCACCCTGA |
| SEQ ID NO.26 | CD37N | CCTCTGGGTCCAAGGGTAGA |
| SEQ ID NO.27 | CD37M | TCTACCCTTAGACCCAGAGG |
| SEQ ID NO.28 | AC2 | TGGCTCACCTGGACAACC |
| SEQ ID NO.29 | AC3 | CCTATCAGAAAGTGGTGGC |
| SEQ ID NO.30 | NC | ACACCAACCGCATCGTCAT |
| SEQ ID NO.31 | CC | CACATCACACACTCTGCGAC |
| SEQ ID NO.32 | Primer βⅠF | ACGGCTGTCATCACTTAGACCTCACCCTGT |
| SEQ ID NO.33 | Primer βⅠR | TCTCCACATGCCCAGTTTCTATTGGTCTCCTT |
| SEQ ID NO.34 | Primer βⅡF | TGATAGGCACTGACTCTCTCTGCCTATTGGTCT |
| SEQ ID NO.35 | Primer βⅡR | TCTGTTTCCCATTCTAAACTGTACCCTGTTAC |
| SEQ ID NO.36 | Primer βⅢF | CCCTAATCTCTTTCTTTCAGGGCAATAATG |
| SEQ ID NO.37 | Primer βⅢR | TTGGACAGCAAGAAAGCGAGCTTAGTGATACTTG |
