# Supplementary material for: A direct multiplex isothermal amplification-reverse dot blot hybridization system for β-thalassemia diagnosis
Source: Ann Hematol. 2025 Nov 18;104(12):6147–59. doi: 10.1007/s00277-025-06711-5 (PMC12764633; doi:10.1007/s00277-025-06711-5)
Supplement: Supplementary file 2 — (DOCX 16.2 KB) [file 277_2025_6711_MOESM2_ESM.docx]

**Table S2: The names of the 17 types of β-thalassemia mutations , as well as the relationship between the mutation site probes and the wild-type site probes.**

| HGVS  nomenclature | site name | mutation type | wild-type site: “N” | Mutation site:  “M” |
| --- | --- | --- | --- | --- |
| HBB:c.126_129delCTTT | CD41-42 | -CTTT | 41-42N | 41-42M |
| HBB:c.130G＞T | CD43 | GAG＞TAG |  | 43M |
| HBB:c.316-197C＞T | IVS-Ⅱ-654 | C＞T | 654N | 654M |
| HBB:c.-78A＞G | -28 | A＞G | -28N | -28M |
| HBB:c.-79A＞G | -29 | A＞G |  | -29M |
| HBB:c.-80T＞C | -30 | T＞C |  | -30M |
| HBB:c.-82C＞A | -32 | C＞A |  | -32M |
| HBB:c.216_217insA | CD71-72 | +A | 71-72N | 71-72M |
| HBB:c.79G＞A | CD26(βE) | GAG＞AAG | βEN | βEM |
| HBB:c.84_85insC | CD27/28 | +C |  | 27-28M |
| HBB:c.94delC | CD31 | -C | 31N | 31M |
| HBB:c.92+1G＞T | IVS-Ⅰ-1 | G＞T | IVS-Ⅰ-1N | IVS-Ⅰ-1M |
| HBB:c.92+5G＞C | IVS-Ⅰ-5 | G＞C |  | IVS-Ⅰ-5M |
| HBB:c.-11_-8delAAAC | Cap+40-43 | -AAAC | CAPN | CapM |
| HBB:c.2T＞C | Initiation codon | ATG＞AGG |  | IntM |
| HBB:c.315+5G＞C | IVS-Ⅱ-5 | G＞C | IVS-Ⅱ-5N | IVS-Ⅱ-5M |
| HBB:c.113G＞A | CD37 | TGG＞TAG | CD37N | 37M |
|  | BC | Blank control | |  |
|  | CC | Color development control | |  |
|  | NC | Negative control | |  |
|  | AC2 | Positive control | |  |
|  | AC3 |  |  |  |

Note: Considering the size of the membrane chip, we have adopted more concise abbreviations for the naming of site information on the membrane chip when making the membrane strips.
