# Supplementary figures and images for: A direct multiplex isothermal amplification-reverse dot blot hybridization system for β-thalassemia diagnosis
Source: Ann Hematol. 2025 Nov 18;104(12):6147–59. doi: 10.1007/s00277-025-06711-5 (PMC12764633; doi:10.1007/s00277-025-06711-5)

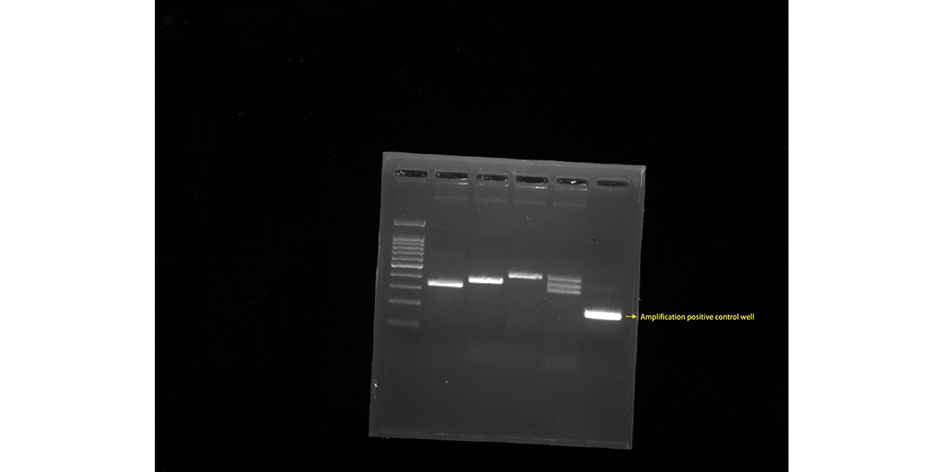

Supplement: Supplementary file 3 — (PNG 136 KB) [file 277_2025_6711_Fig6_ESM.png]

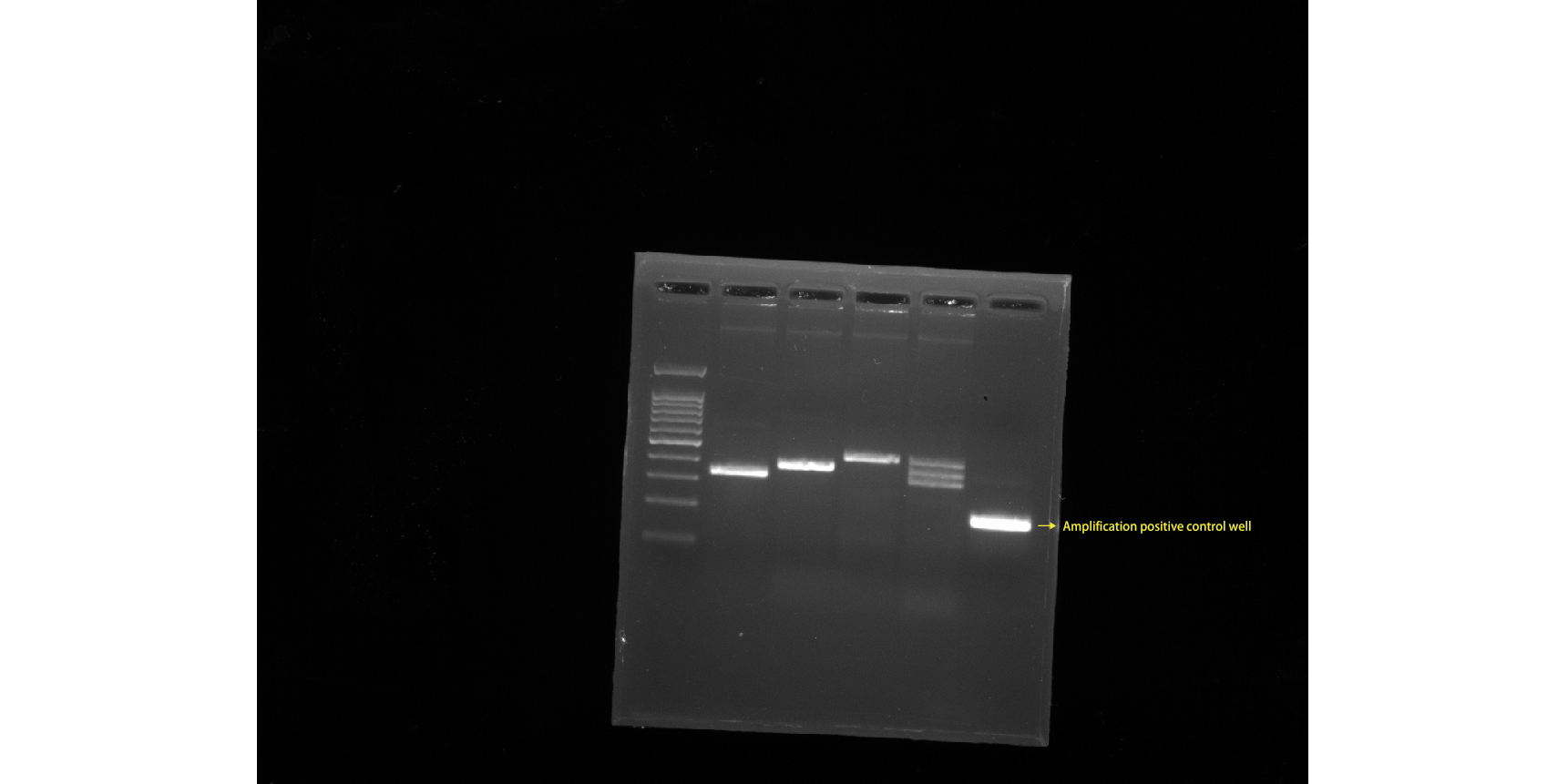

Supplement: Supplementary file 4 — High Resolution Image (TIF 7.06 MB) [file 277_2025_6711_MOESM3_ESM.tif]

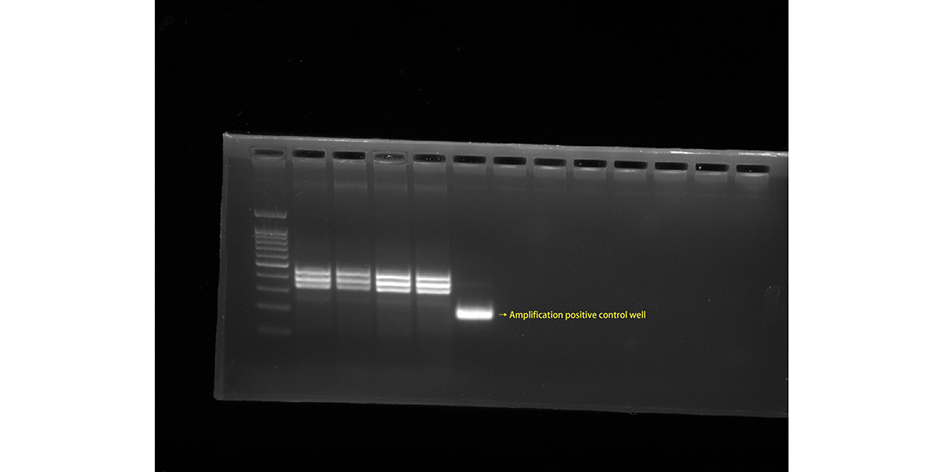

Supplement: Supplementary file 5 — (PNG 149 KB) [file 277_2025_6711_Fig7_ESM.png]

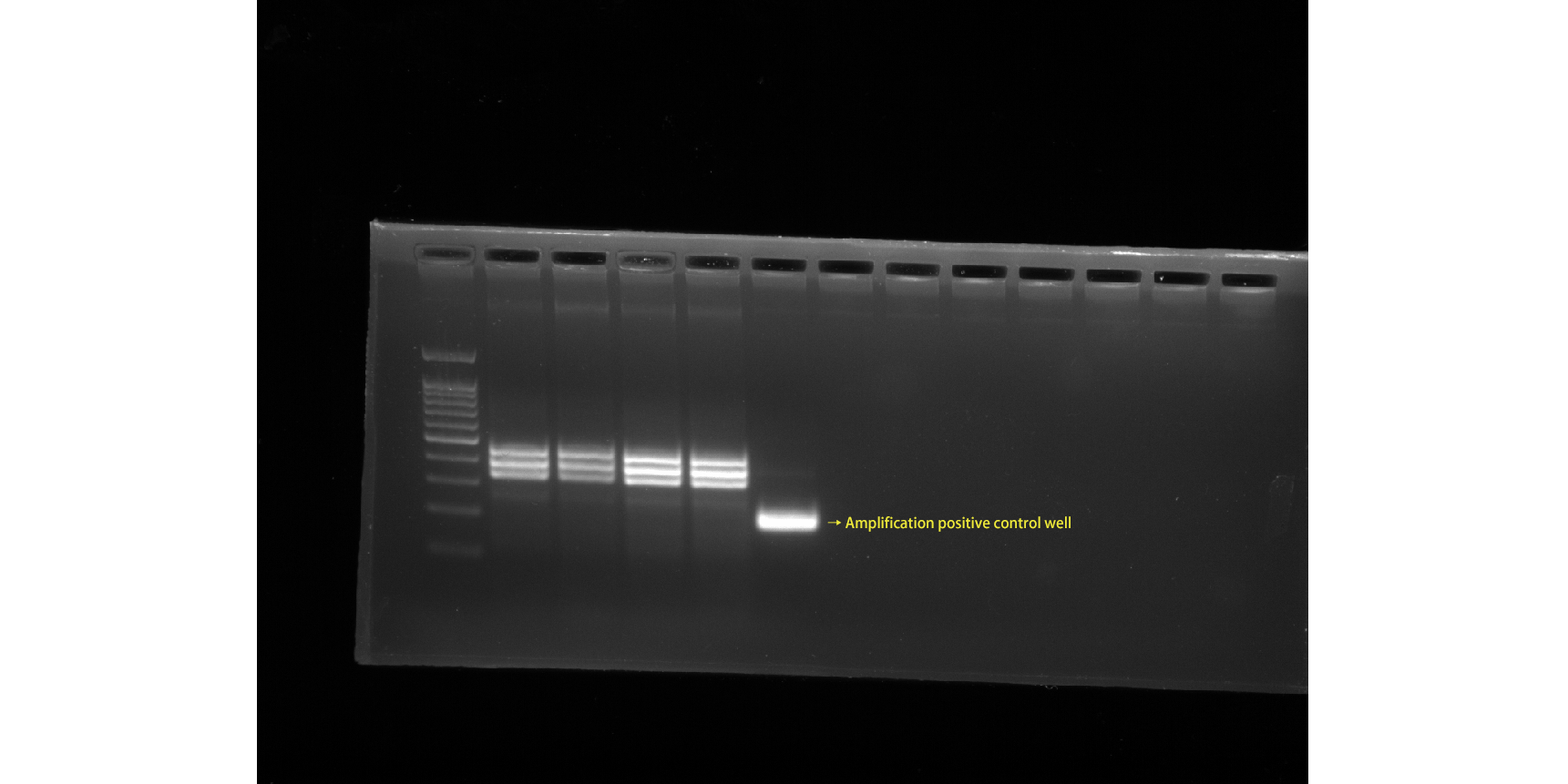

Supplement: Supplementary file 6 — High Resolution Image (TIF 7.11 MB) [file 277_2025_6711_MOESM4_ESM.tif]

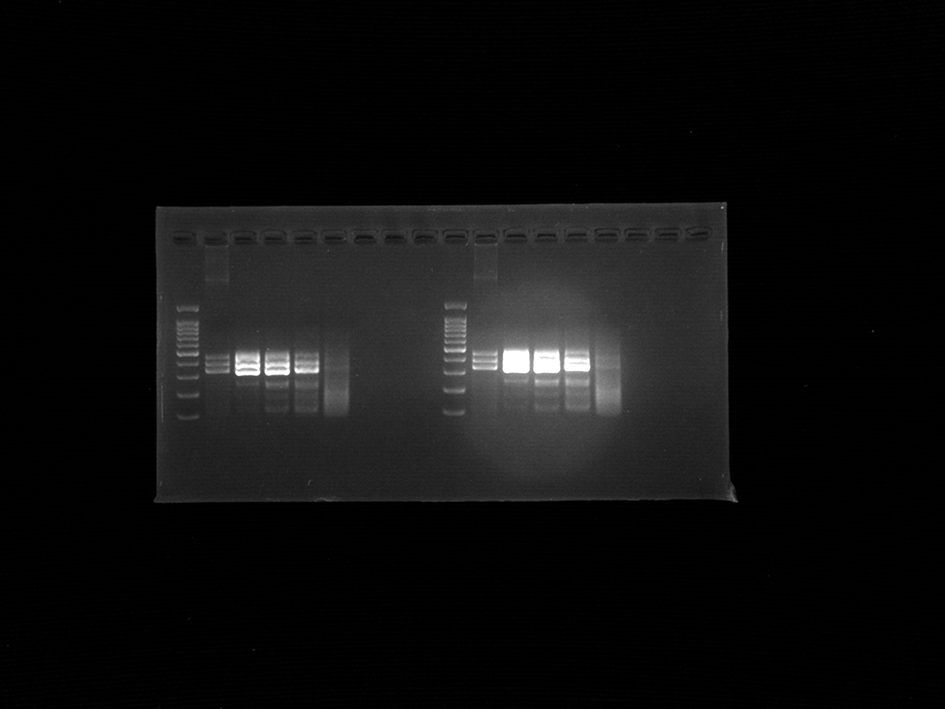

Supplement: Supplementary file 7 — (PNG 249 KB) [file 277_2025_6711_Fig8_ESM.png]

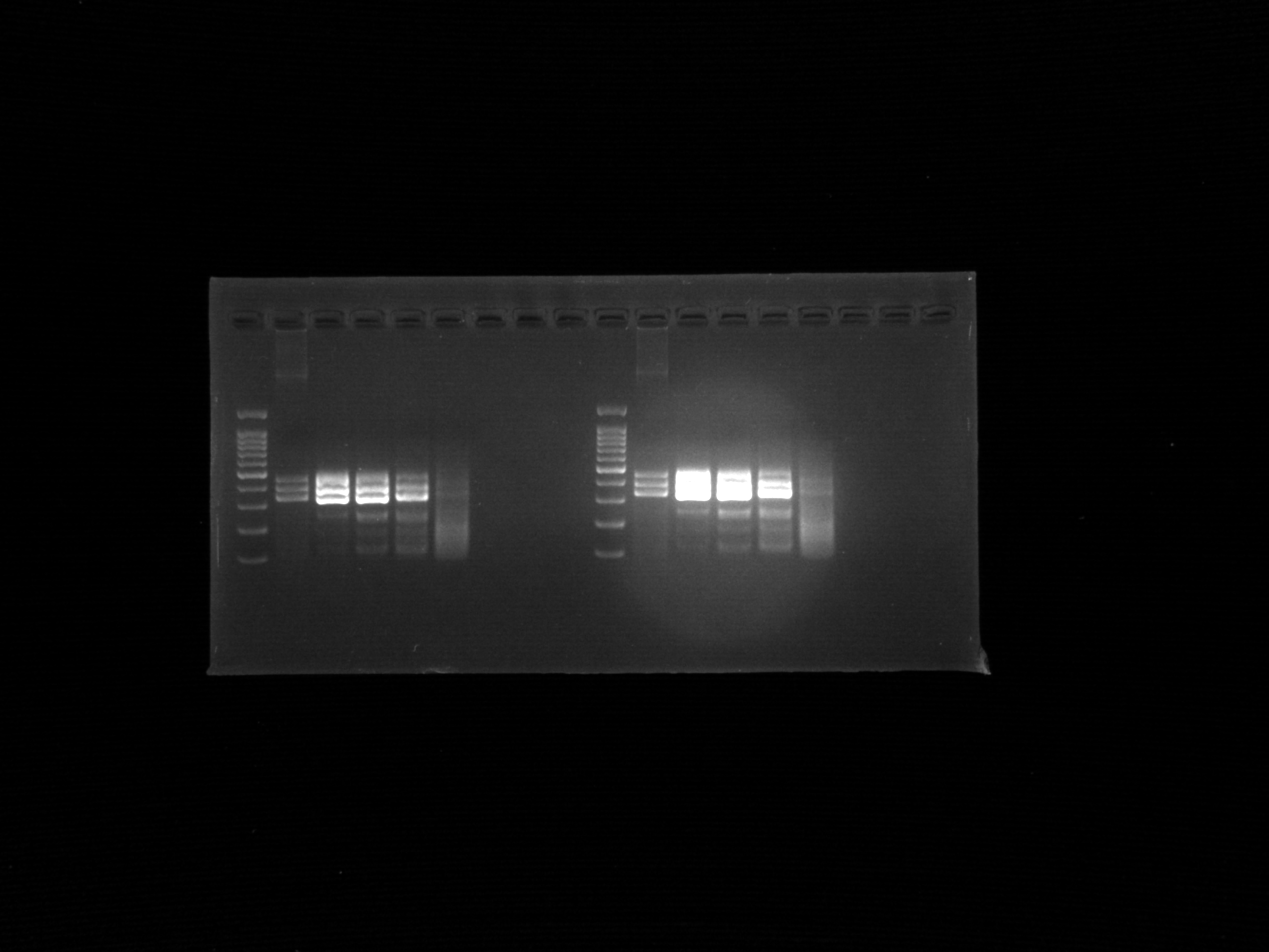

Supplement: Supplementary file 8 — High Resolution Image (TIF 15.5 MB) [file 277_2025_6711_MOESM5_ESM.tif]

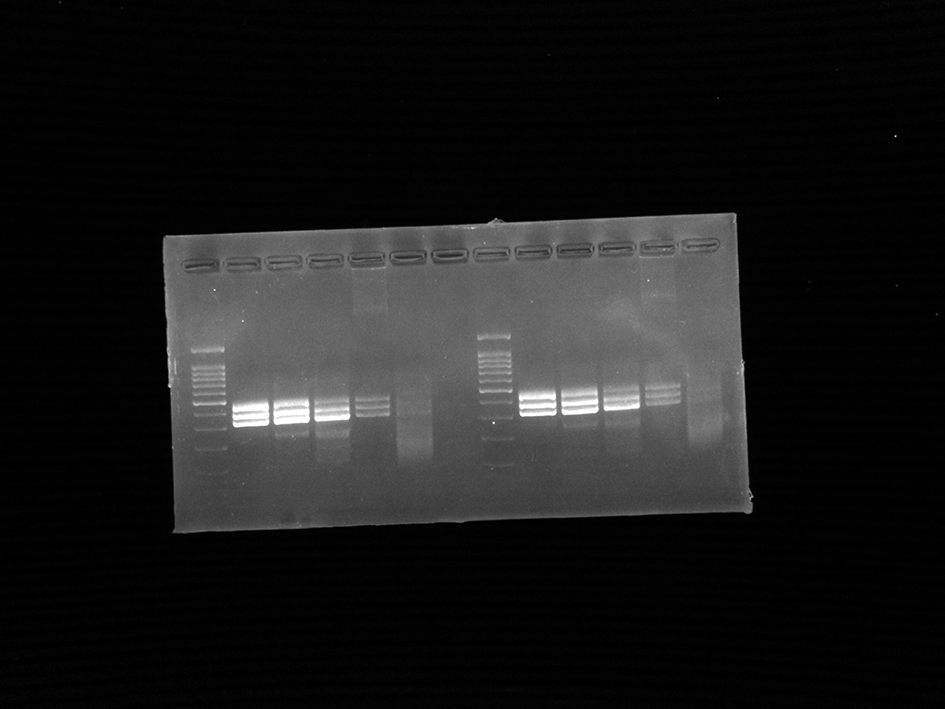

Supplement: Supplementary file 9 — (PNG 285 KB) [file 277_2025_6711_Fig9_ESM.png]

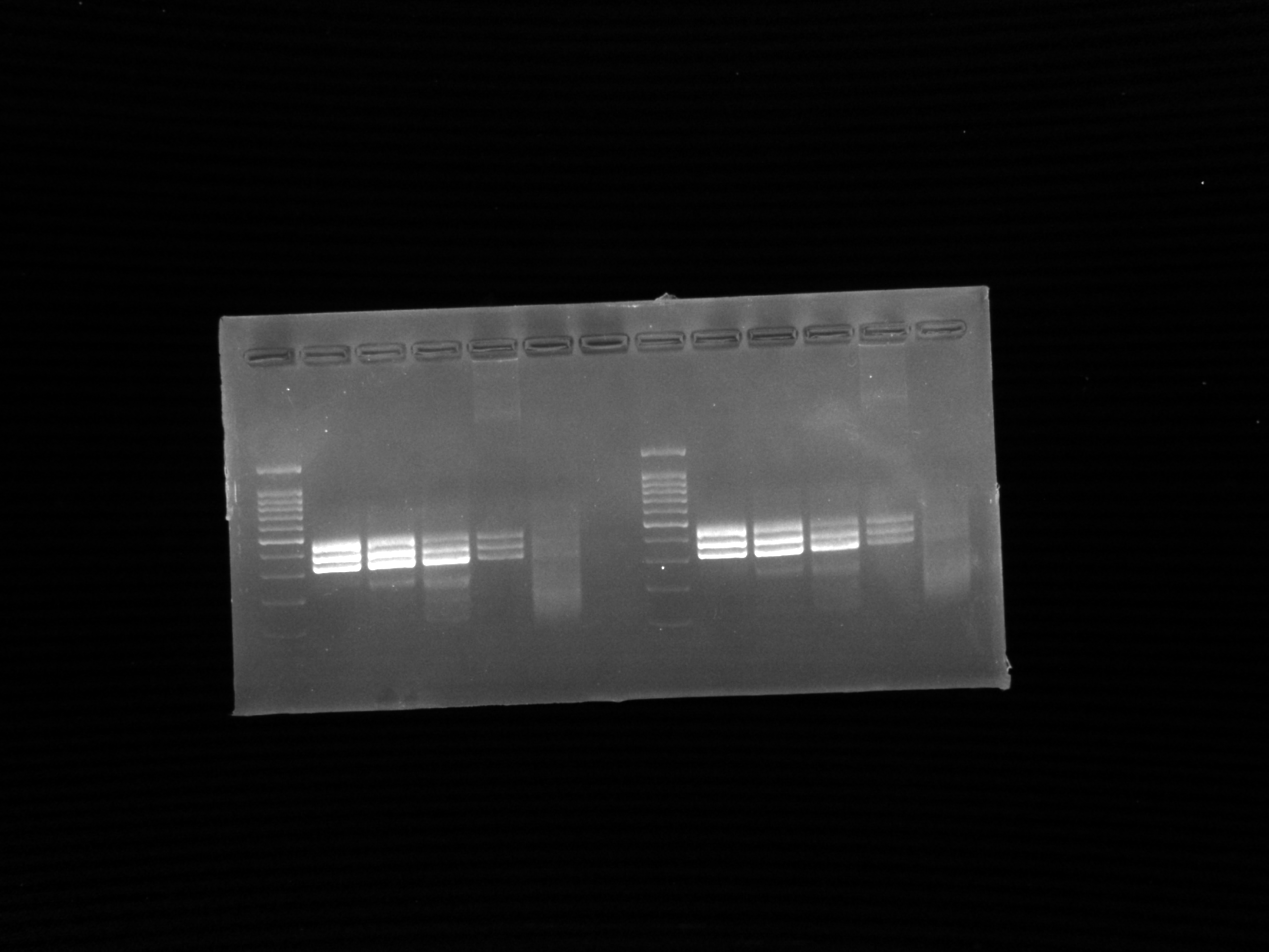

Supplement: Supplementary file 10 — High Resolution Image (TIF 15.5 MB) [file 277_2025_6711_MOESM6_ESM.tif]

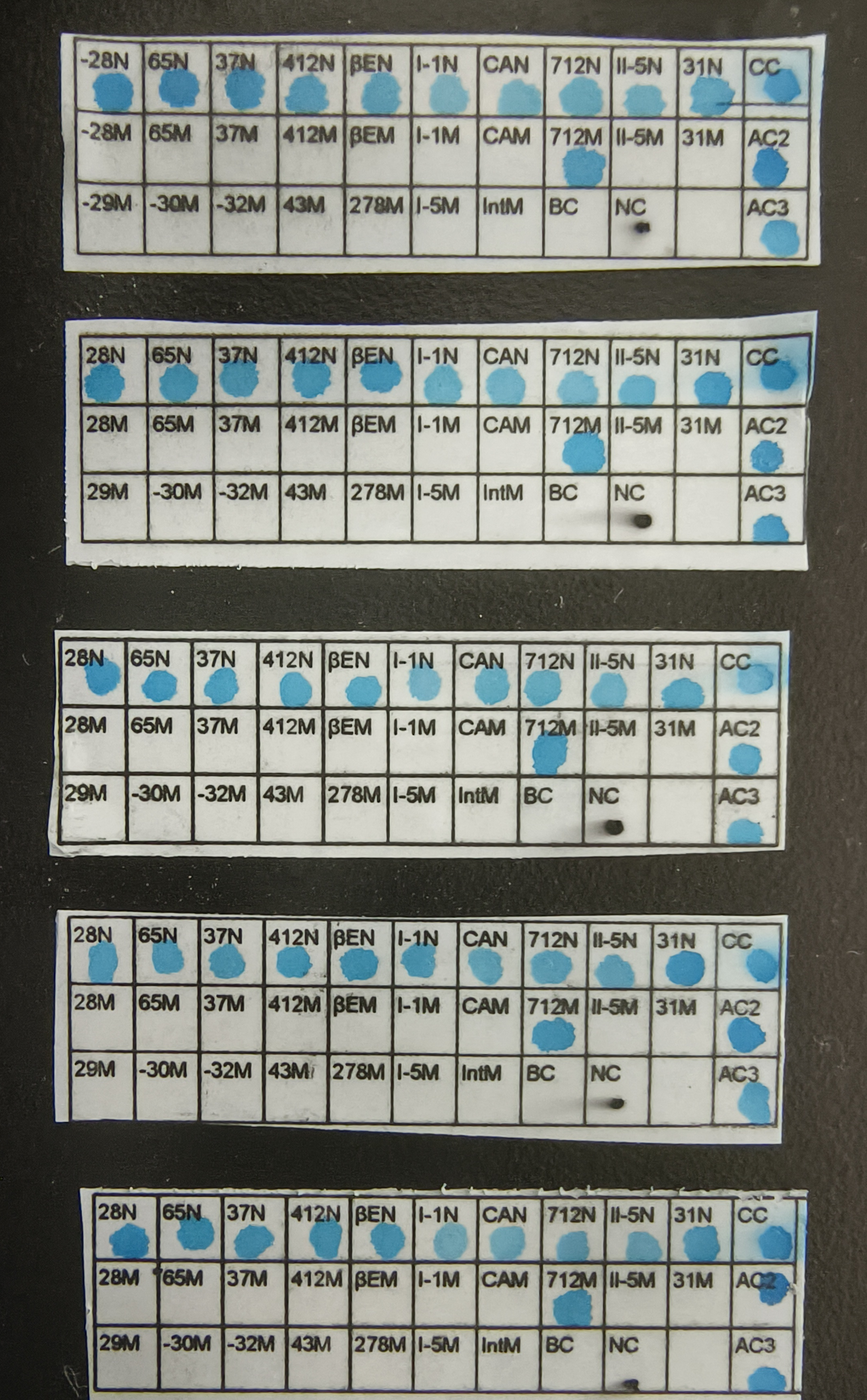

Supplement: Supplementary file 11 — (PNG 7.42 MB) [file 277_2025_6711_MOESM7_ESM.png]

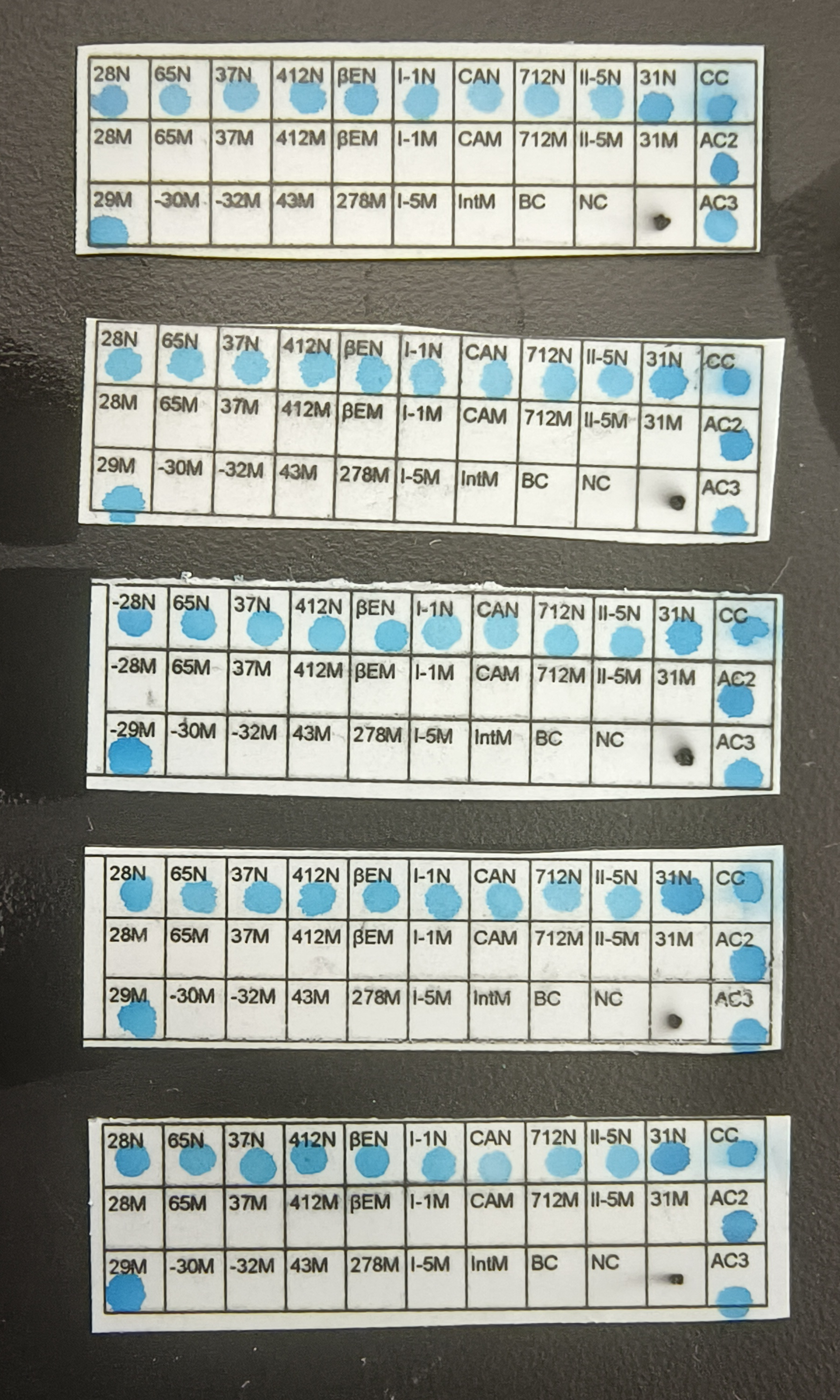

Supplement: Supplementary file 12 — (PNG 5.66 MB) [file 277_2025_6711_MOESM8_ESM.png]

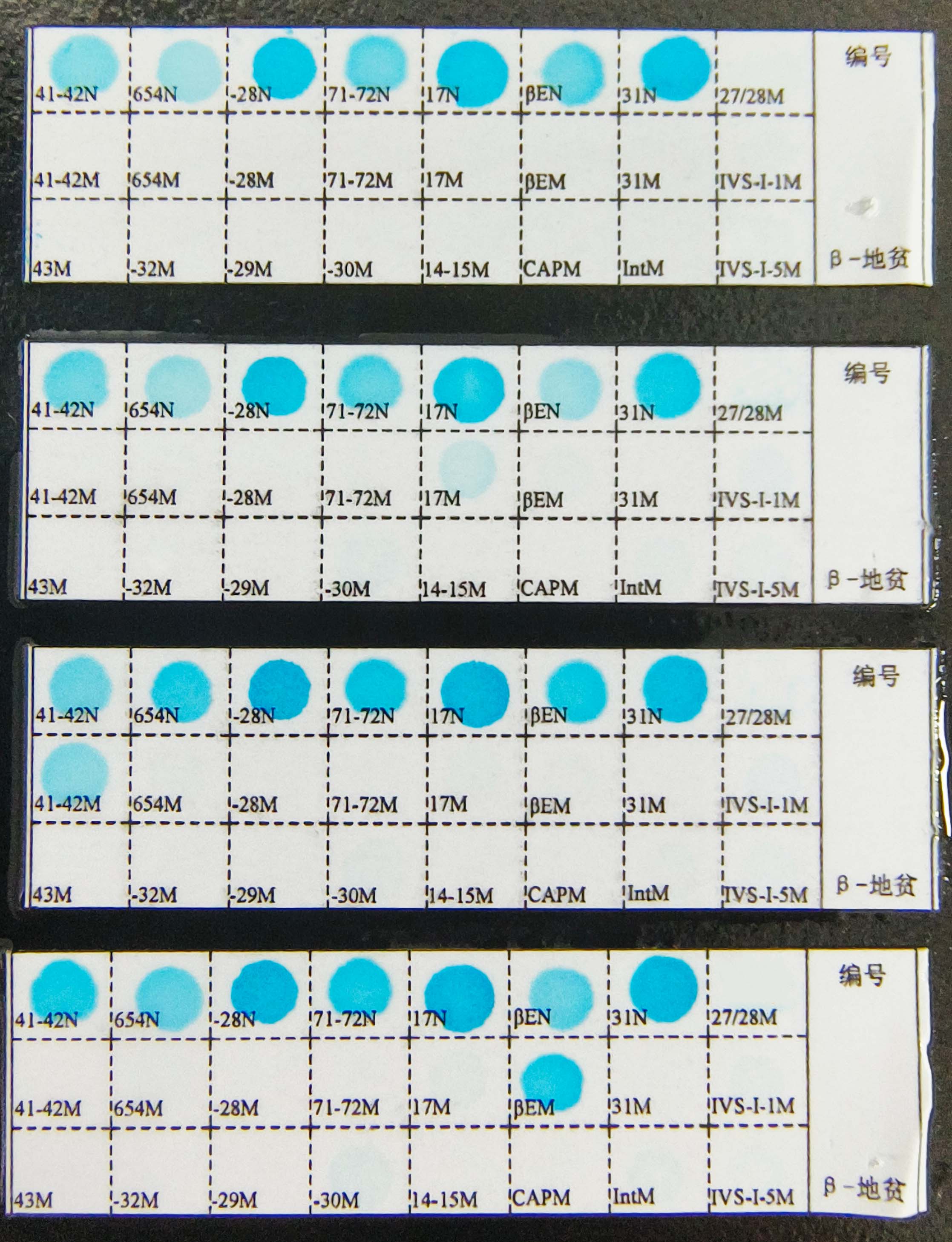

Supplement: Supplementary file 13 — (JPG 448 KB) [file 277_2025_6711_MOESM9_ESM.jpg]
